# Patient Empowerment in the Context of Outpatient Surgery Using the Example of Orthopedics (Power-AOP): Protocol for a Mixed Methods Study

**DOI:** 10.2196/87249

**Published:** 2026-04-27

**Authors:** Godwin Denk Giebel, Gregor Max Giebel, Danko Milinkovic, Carina Abels, Hanns Richard Cissarek, Carsten Perka, Sophia Zander, Udo Schneider, Mechthild Kern, Bernd Kladny, Orkan Okan, Eva Maria Bitzer, Jürgen Wasem, Theresa Hüer, Frederik Valbert

**Affiliations:** 1 Institute for Health Care Management and Research, University of Duisburg-Essen, Thea-Leymann-Str. 9, Essen, North Rhine-Westphalia, 45127, Germany, 49 201183 ext 3180; 2 Center for Musculoskeletal Surgery, Charité - Universitätsmedizin Berlin, Berlin, Germany; 3 Techniker Krankenkasse, Hamburg, Germany; 4 Das PatientenForum e.V, Bubenheim, Germany; 5 German Society for Orthopaedic and Trauma Surgery, Berlin, Germany; 6 Deutsches Netzwerk Gesundheitskompetenz, Köln, Germany

**Keywords:** orthopedics, patient empowerment, ambulatory surgery, outpatient surgery, orthopedic surgery, study protocol, patient-centered care

## Abstract

**Background:**

Shifting surgeries from the stationary to the outpatient setting is seen as a suitable way to increase efficiency in the health care system. A substantial increase in outpatient procedures can therefore be observed internationally—particularly in the field of orthopedics. However, the interests and needs of patients are often insufficiently taken into account in this process. The “Power-AOP” research project was initiated to identify the associated challenges in the area of patient empowerment and to develop solutions.

**Objective:**

Using the field of orthopedics as an example, the Power-AOP project investigates how patient empowerment can be strengthened in the context of outpatient surgery.

**Methods:**

Using a mixed methods approach, health policy recommendations will be developed that aim to strengthen patient empowerment in the context of outpatient surgery. The project is scheduled to run for 3 years and comprises 6 work packages, with a total of 10 modules. In the first step, a scoping review was carried out to map the existing literature. This is followed by focus groups and interviews with patients and health care providers to gain deeper insights into their experiences and perspectives. The results will be quantified using a questionnaire-based survey. In order to identify a suitable patient population for this survey, an analysis of claims data will be conducted beforehand. The results will then be discussed and refined in 2 stakeholder workshops with key players in the health care system. In the final phase, a concept will be developed that contains actionable recommendations for strengthening patient empowerment in the context of outpatient care.

**Results:**

The project began in April 2025. The claims data analysis and the scoping review have been completed, and a manuscript for the review is currently being prepared. The focus groups and interviews were conducted with 19 health care providers and 26 patients. Data analysis is in progress. The quantitative survey is planned for May 2026 to March 2027. The workshops are scheduled for the second and fourth quarters of 2027. The project will conclude with the final results in March 2028.

**Conclusions:**

This project will help to improve patient empowerment in the context of outpatient surgery. This allows patients to take a more active role in the process. On the one hand, this can lead to greater satisfaction with the process, particularly among patients. On the other hand, more active participation by patients can improve outcomes and prevent unnecessary readmissions or additional treatments.

## Introduction

Internationally, the number of surgical procedures carried out as an outpatient service has increased markedly in Organization for Economic Co-operation and Development countries. This is especially evident in cases of cataract surgery and tonsillectomy [1]. Regarding cataract surgery, the share of cases increased from 46% in 2011 to 91% in 2021 in Austria and from 35% to 77% in Hungary [1]. The proportion of tonsillectomies performed as outpatient cases increased from 45% in 2011 to 80% in 2021 in Sweden and from 39% to 70% in the United Kingdom [1]. Besides improving patient safety and health outcomes, outpatient surgeries can shorten treatment episodes and thereby save important resources [1]. This potential is also acknowledged in the German health care system. The National Association of Statutory Health Insurance Physicians estimates that 3 to 4 million of the 16 million in-patient surgeries in Germany could be carried out on an outpatient basis. This would result in savings of several billion euros [2].

A high proportion of outpatient surgeries stems from the field of orthopedics. Thus, a study on major outpatient surgeries performed at hospital-owned facilities in the United States found that surgeries related to the musculoskeletal system accounted for 7 of the top 20 outpatient surgery categories and 22% of all major outpatient surgeries in 2019 [3]. In Germany in 2022, orthopedics ranked second in the number of outpatient surgeries performed, accounting for 14.7% of all surgeries [4]. In addition to their frequency, outpatient surgeries are characterized by the fact that the patients affected represent a broad cross-section of the population in terms of age, sex, and socioeconomic status [3].

Despite the potential benefits and high relevance of outpatient (orthopedic) surgeries, they are also associated with several problems [5]. These issues arise, for example, in the context of limited information (eg, optimal activity level) and the psycho-social domain (eg, difficulties in obtaining practical and emotional support from either their families and friends or professionals) [6].

Two important issues should be highlighted. First, patients have poor postoperative recall of information provided on the day of surgery. This was confirmed by Shultz et al [7], who found that 21.9% did not remember the postoperative conversation at all and that 75.2% rated their memory as very poor or poor. Second, dealing with pain and analgesics can be challenging for patients [8].

To face such and other challenges, improving patient empowerment (PE) can be a suitable approach. Generally, PE is associated with better patient outcomes such as quality of life, improved clinical outcomes (eg, health status), and independence from health care providers and caregivers [9]. Particularly, in the field of outpatient orthopedic surgeries, PE plays a pivotal role, as freedom of movement is often restricted and patients are often dependent on analgesics.

In line with these problems and with the conviction that improving PE can sustainably improve care, the project “Patient Empowerment in the Context of Outpatient Surgery Using the Example of Orthopedics (Power-AOP)” investigates how PE can be improved in the context of outpatient surgery. Based on the relevance of orthopedics in this context as described above, we have chosen this as an example for our project.

## Methods

### Study Design

The study aims to develop recommendations to promote PE in the context of outpatient surgeries. To achieve this goal, 6 overarching research questions will be investigated (Textbox 1).

Textbox 1. **Research questions.**

Research questions

- 1. What challenges and hurdles for patients stand in the way of optimal planning, implementation, and follow-up care for outpatient operations?
- 2. What measures should be used before and after outpatient surgery to improve patient empowerment, and how should these be structured?
- 3.1. How do patients assess the relevance of the problems identified in research question 1?
- 3.2. Which measures from research question 2 are considered by patients to be particularly important?
- 3.3. Where are differences between patient groups in research questions 3.1 and 3.2?
- 4. How should the findings be incorporated into (health policy) recommendations for patient empowerment in the context of outpatient surgery in general and specifically for orthopedics?

**Figure 1.**
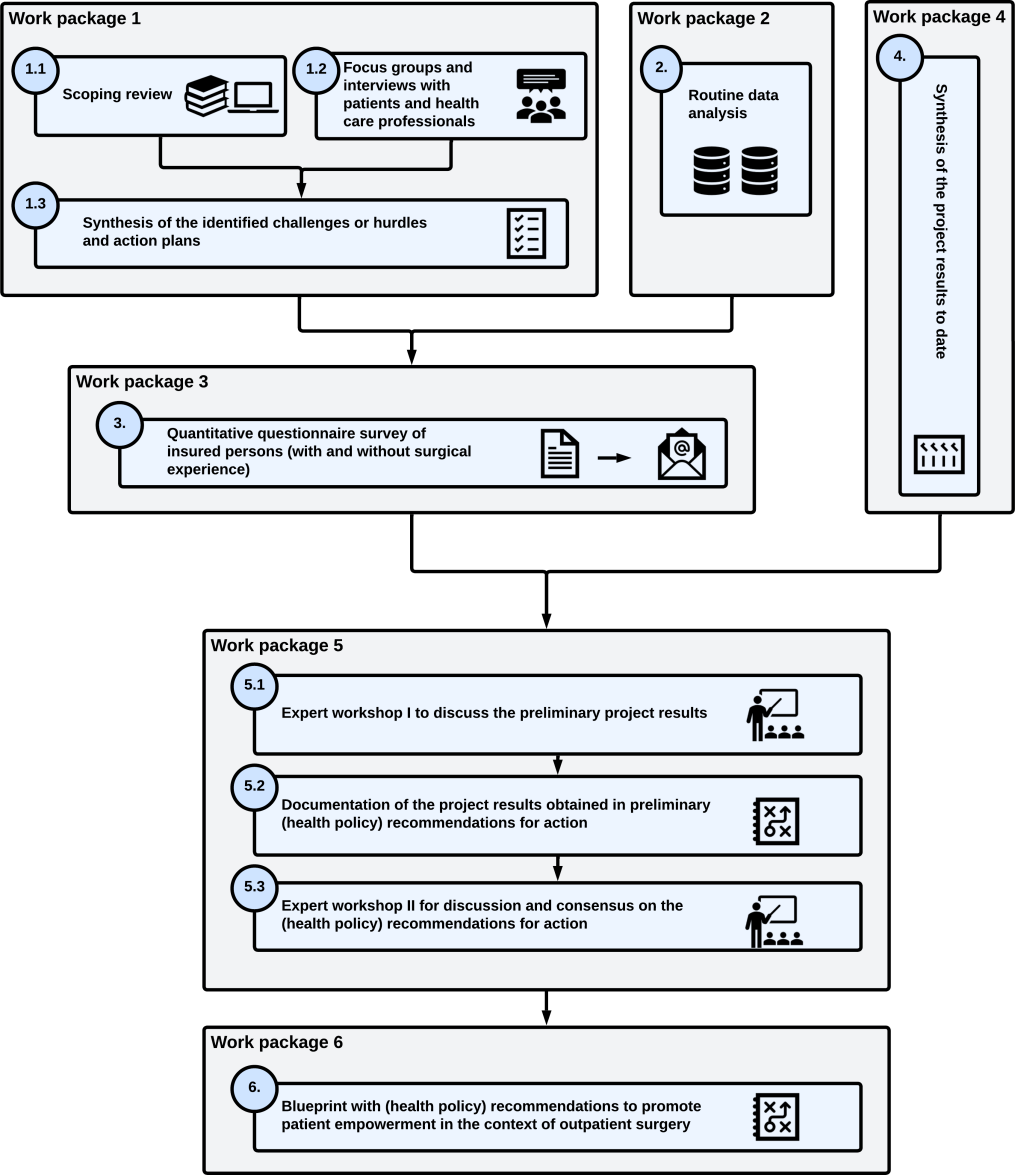
Study design represented in a flowchart.

A mixed methods design is used to reach this goal. The distinct approach is visualized in Figure 1. The study is being conducted from April 2025 to March 2028.

The mixed methods design not only aims to use different research methods, such as reviews and qualitative and quantitative elements, but also aims to connect and integrate them. This is ensured by using the (preliminary) results of respective work packages as a starting point for subsequent work packages.

The study began with an exploratory investigation of challenges as well as corresponding solutions in the context of outpatient surgeries (work package 1). Therefore, a scoping review (module 1.1) as well as focus groups and interviews with patients and health care professionals (module 1.2) were conducted. At the same time, an analysis of claims data was performed (work package 2), aiming to identify relevant criteria that will allow the selection of eligible patients for the quantitative survey in the subsequent work package. The patient group determined in work package 2 will be surveyed in work package 3 to ascertain the relevance of the aspects identified in work package 1. As part of a continuous process, the preliminary project results will be synthesized to guarantee an optimal integration between literature, qualitative, and quantitative research methods (work package 4). Work package 5 serves to discuss the results obtained up to that point in a workshop with experts (module 5.1). The workshop results will then be used to develop preliminary recommendations to improve PE in the context of outpatient surgery (module 5.2). Subsequently, these will be discussed and consented to in a second workshop (module 5.3). Finally, in work package 6, the recommendations that have been approved will be documented in a blueprint and subsequently translated into a format that is comprehensible to patients. An essential part of the blueprint will include the recommendations concerning the targeted design and dissemination of informational materials intended for patients. A more detailed description of each work package and module will be provided subsequently.

### Project Basis (Work Package 1)

The project began with an exploratory investigation of the challenges that patients face in the context of outpatient (orthopedic) surgery (research question 1) and the corresponding improvement in PE (research question 2).

#### Scoping Review (Module 1.1)

The first step was an extensive scoping review with 2 parallel (selection and evaluation) arms: a first arm to identify patient-side challenges in the context of outpatient surgeries (research question 1) and a second arm to collect possible ways to improve PE in this context (research question 2). The scoping review follows the methodology described by Arksey and O’Malley [10] as well as by Levac et al [11]. Its objective is 2-fold: to provide an overview of the available evidence in the literature and to identify existing evidence gaps. The literature search was led by the Centre for Musculoskeletal Surgery, Charité-University Medical Center Berlin, and was conducted together with the Institute for Health Care Management and Research, University of Duisburg-Essen.

The literature search is based on medical databases (MEDLINE via PubMed and Embase). The PCC scheme (Population=all parties involved in the operation process; Concept=challenges and hurdles and measures to overcome them; and Context=outpatient orthopedic operations) was used to develop the search strategy. Boolean operators and eligible keywords that were identified in preliminary searches were used to operationalize the search. The final search strings are provided in MultimediaAppendices 1  2. Inclusion and exclusion criteria were determined and are presented as Textboxes2  3.

Textbox 2. **Inclusion criteria.**

- Problems and barriers as well as solutions and potential for optimization concerning the patient empowerment in the context of outpatient orthopedic surgery
- Articles published in one of the databases investigated
- Language: English or German

Textbox 3. **Exclusion criteria.**

- No distinct focus on patient empowerment
- No explicit or exclusive focus on outpatient surgery
- No explicit or exclusive focus on orthopedic surgery
- Focus on pediatric care
- Corresponding full-text article was available and included
- No full-text available
- Conference abstract
- Language other than English or German

**Figure 2.**
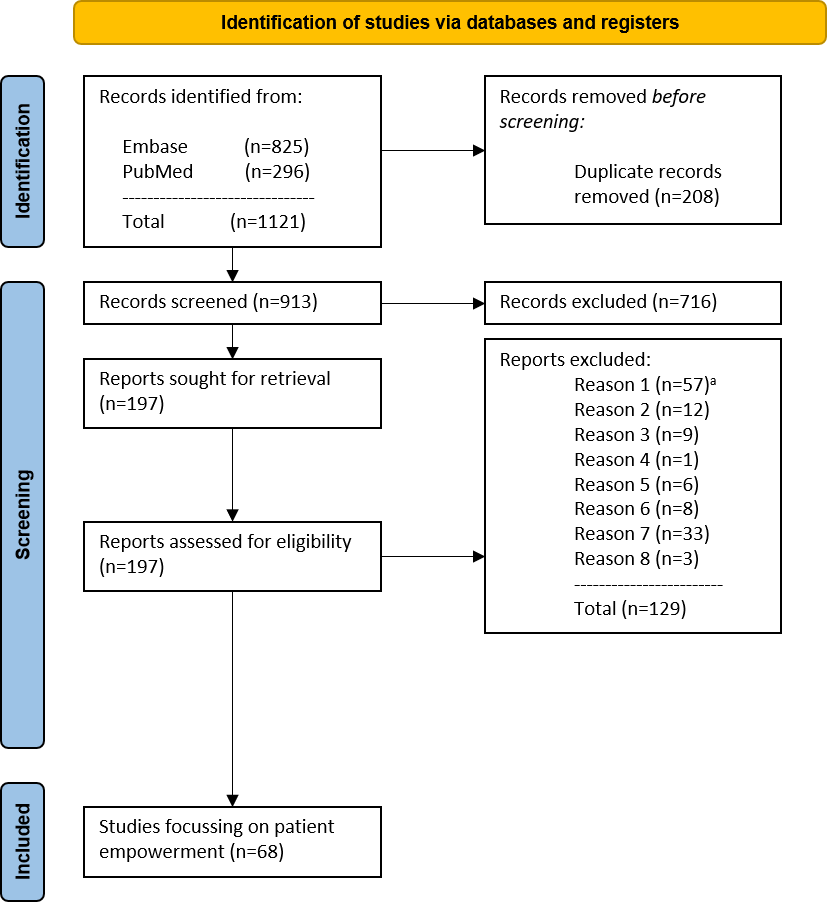
PRISMA (Preferred Reporting Items for Systematic Reviews and Meta-Analyses) flow diagram of the study selection process. ^a^Reason 1: no distinct focus on patient empowerment; reason 2: no explicit or exclusive focus on outpatient surgery; reason 3: no explicit or exclusive focus on orthopedic surgery; reason 4: focus on pediatric care; reason 5: corresponding full-text article was available and included; reason 6: no full-text available; reason 7: conference abstract; and reason 8: language other than English or German

Two reviewers independently used these criteria to assess the literature in a 2-stage (title and abstract as well as full text) screening process. The screening process is presented in Figure 2.

Finally, the identified literature was categorized into three domains: (1) relevant to research question 1 (challenges), (2) relevant to research question 2 (improvement of PE), and (3) neither relevant to research question 1 nor to research question 2 and subsequently excluded. A double assignment to groups 1 and 2 was possible. In addition to conducting database research, a structured search was also carried out on the websites of relevant stakeholders, particularly associations and public institutions.

Assuming high heterogeneity of the results, the included articles were subjected to a qualitative content analysis. First, the included articles were analyzed and coded using suitable software (MAXQDA; VERBI Software GmbH). Then, the relevant identified text passages were discussed and systematized within an internal workshop. The results of both arms are captured and published separately. To guarantee a structured process and reporting, the JBI Manual for Evidence Synthesis [12], as well as the PRISMA-ScR (Preferred Reporting for Systematic Reviews and Meta-Analysis Extension for Scoping Reviews), were used [13].

#### Focus Groups and Interviews With Patients and Health Care Professionals (Module 1.2)

The results of the scoping review were discussed in focus groups and interviews with patients and health care professionals and were supplemented with different positions and perspectives. In total, up to 6 focus groups, each with a maximum of 10 participants, were planned. As recruitment did not allow for such a composition, individual or double interviews were supplemented to fully reflect the perspectives. Focus groups and interviews were conducted until key statements were frequently repeated. Half of the conversations were planned to be held with patients who have suffered a meniscus tear (either operated on or not), and the other half were planned with health care professionals. Patients included (1) inpatient surgical patients, (2) outpatient surgical patients, and (3) patients with the diagnosis who have not yet undergone surgery but for whom surgery might be indicated. Focus groups and interviews were supplemented by relatives of the patients. Health care professionals were required to work in the context of outpatient surgery. They include outpatient orthopedic surgeons, doctors in private practices, doctors working in hospitals, and other practitioners, such as caregivers or physiotherapists.

The focus groups and interviews were held online using a meeting platform (Zoom). Contact was mainly initiated personally by the Centre for Musculoskeletal Surgery, Charité-University Medical Center, Berlin, as well as the German Society for Orthaopedics and Trauma. The University of Duisburg-Essen provided support with establishing contact. Subsequent recruitment was conducted by the University of Duisburg-Essen.

The focus groups and interviews are under the responsibility of the Institute for Health Care Management and Research, University of Duisburg-Essen. They were moderated by an interdisciplinary team using a semistructured guideline, building upon the preliminary findings of the scoping review and optimized through discussion within the team. The guidelines were pretested in advance by medical professionals and members of the PatientForum e.V. Since the guideline served only as a framework for the interviews, it enabled the moderators to capture emerging themes while maintaining consistency in the core questions across all groups. The discussions were recorded and subsequently transcribed. Transcripts are currently subjected to a content-structuring qualitative content analysis based on Mayring [14] and Kuckartz [15]. Coding is done with deductive and inductive codes and quality assured according to the dual control principle. The results of the focus groups and interviews will be published.

#### Synthesis of the Preliminary Results (Module 1.3)

At the end of work package 1, the preliminary results will be summarized and systematized by the Institute for Health Care Management and Research, University of Duisburg-Essen. Therefore, a matrix will be developed that contains the various problems and solutions identified in each module. This includes both the identified challenges and the ways to improve PE. The summary will serve as a starting point for work package 3.

### Claims Data Analysis (Work Package 2)

The second work package aims to ascertain the target population for work package 3. An orthopedic indication example will be determined based on several criteria. The selected example must be quantitatively relevant, frequently lead to surgery (in an outpatient setting), and represent a broad sociodemographic spectrum of insured individuals. To this end, claims data from the Techniker Krankenkasse (TK) are used. The TK is the largest statutory health insurance fund in Germany, covering over 11 million insured people.

Initially, the consortium identified relevant orthopedic *International Classification of Diseases, Tenth Revision* (*ICD-10*) codes and evaluated various procedures to validate the diagnosis in the claims data. After agreeing on which validation method appeared most suitable for each potential indication in terms of specificity and sensitivity, the various potential cohorts were compared across a number of relevant parameters. Possible parameters included the number of cases within 1 year, frequency distribution within 1 year, frequency distribution between different sociodemographic groups (such as sex or age), frequency of OPS-Codes (procedure classification for the encoding of operations, procedures, and general medical measures) that indicate a surgical therapy, and average time between first diagnosis and operation as well as the sector in which the surgical therapy took place. Preferably, the corresponding procedure should be listed in the “catalog of operations that can be performed on an outpatient basis” applicable in Germany [16]. Potential indications include, among others, meniscus injuries, carpal tunnel syndrome, and cartilage damage in the knee or lower leg.

### Quantitative Survey (Work Package 3)

A standardized, written, retrospective survey with insured persons of the TK is conducted in work package 3. It aims to assess the identified challenges and solutions from work package 1 from the perspective of different groups of patients (eg, grouped by sociodemographic or socioeconomic characteristics or experience with outpatient surgeries; research question 3). Challenges and solutions should not only be considered independently but also in relation to each other.

The survey includes patients with the indication determined in work package 2. In the first step, a random sample of insured persons with the determined indication (both operated or not) will be drawn by the TK. A filter question at the beginning of the survey will be used to identify if a respondent has already undergone surgery, and if so, in which setting. The length of the selection period depends on the frequency of selected ICD-Codes in the TK data as well as potential seasonality. Further selection details will originate from work package 2.

The questionnaire, the corresponding information letter, and the consent form will be developed by the Institute of Health Care Management and Research, University of Duisburg-Essen, in close cooperation with the medical partner, the TK, and the patient representatives. The pilot questionnaire will be subjected to a pretest using think-aloud and probing [17].

Selected TK policyholders will be contacted in 1 of 2 ways, depending on whether they have activated their (digital) TK mailbox. If activated, the invitation will be sent digitally; otherwise, it will be sent by mail. The letter contains a QR link that takes participants to a website of the TK containing detailed information about the survey and inviting them to take part. The actual survey is accessed via a separate link that leads to an established online survey tool (eg, LimeSurvey) hosted by the University of Duisburg-Essen.

Based on experience from previous projects in which patients were surveyed, a response rate of 10% is assumed. A reminder is planned to ensure a sufficiently large number of respondents.

In principle, the analyses conducted in work package 3 are descriptive in nature, focusing on metrics such as frequencies, averages, or median values. The primary end point is the assessment of challenges and corresponding solutions identified in work package 1, which can be measured, for example, using a 10-point Likert scale. Additionally, inductive statistics are used to test the significance of differences between groups. The evaluation will be conducted by the Institute of Health Care Management and Research, University of Duisburg-Essen, using eligible statistical software (such as Visual Studio Code, Microsoft; SPSS Statistics, IBM; or RStudio, Posit PBC). The results will be interpreted by the whole consortium. The distinct evaluation depends on the results of work packages 1 and 2.

As the study modules build on each other, the questionnaire content (eg, primary end point structure or final survey instrument) depends on the scoping review and qualitative research, and the exact target population for the survey will be determined by the routine data analysis. Therefore, it is not possible to estimate the exact number of cases a priori. Instead, a more generic calculation of the number of cases required to perform potential analyses with sufficient power has been carried out. The power of a nonparametric significance test (Mann-Whitney *U* test) on the differences in the (ordinal) assessment of a primary end point from 2 subgroups (n_1_ and n_2_) was calculated under different assumptions (cf. Multimedia Appendix 3). Assuming the unfavorable circumstances that n_2_ includes only 300 (15%) respondents (the other 1700 (85%) are in n_1_) and the effect size according to Cohen is to be rated as weak (0.2), 2000 respondents were sufficient to reach a power of ≥90% [18]. Given the assumed response rate of 10%, 20,000 insured people should be selected and contacted.

### Synthesis of the Preliminary Project Results (Work Package 4)

The fourth work package serves to merge the results of the quantitative survey (work package 3) with the results of the literature and qualitative research (work package 1). This is a continuous process that already began with the development of the initial findings.

The synthesis is carried out using 2 different approaches. First, the results of individual modules (scoping review, focus groups, and interviews) will be used as a starting point for subsequent modules. Thus, the results of the scoping review serve to develop a guideline for the qualitative survey (focus groups and interviews), and the results of the qualitative survey will be used to conceptualize the questionnaire for the quantitative survey (work package 3). Furthermore, the target population for the quantitative survey will be determined based on the claims data analysis (work package 2). Second, the results of the scoping review, the qualitative results, and the results of the quantitative survey will be compared, systematized, and combined to create a starting point for work package 5, the assessment of the project results.

The results of this work package serve as a starting point for the next one.

### Assessment of the Project Results (Work Package 5)

The fifth work package consists of three modules: (1) expert workshop 1, (2) development of preliminary (health policy) recommendations, and (3) expert workshop 2. It serves to approve the content of the blueprint to be developed in work package 6.

#### Expert Workshop 1 (Module 5.1)

The first workshop aims to discuss the preliminary project results (identified challenges in the context of outpatient surgeries as well as corresponding solutions) with experts to assess their eligibility for (health policy) recommendations (research question 4). Participants will represent the interests of stakeholders involved in the operation process in a broader sense: patient representatives, representatives of associations of hospital-based and office-based surgeons, as well as representatives of statutory health insurance. The workshop will include up to 16 experts, leading to 4 groups each with 4 experts. The workshop will start with input presentations, followed by a “World-Café-Discussion” and the presentation of its results in the plenum.

During the “World-Café-Discussion,” the challenges and corresponding solutions will be discussed on 4 high tables, each staffed with 4 experts and 1 project member. The central content of the discussion will be written on writable tablecloths. After 20 minutes of discussion, the expert groups will switch tables to discuss the next challenge. The project member of each table will explain the previous discussion to the new group, enabling deepening of the thoughts. The “World-Café” will end when each group of experts has visited each high table and discussed each challenge. Subsequently, all results (the paper tablecloths) will be presented in the plenum. Based on a protocol as well as the paper tablecloths, the Institute of Health Care Management and Research, University of Duisburg-Essen, will systematize and summarize the findings.

#### Development of Preliminary (Health Policy) Recommendations (Module 5.2)

Following the first expert workshop, the project consortium translates the results into preliminary (health policy) recommendations. These will be used as a starting point for the second workshop.

#### Expert Workshop II (Module 5.3)

The discussion in the second workshop serves to finalize the preliminary (health policy) recommendations and to adapt them according to the expert opinions. A maximum of 16 experts will participate, emerging from the same fields as the experts in the first workshop. The second workshop will be held online and will be supported by a voting tool (eg, Microsoft Forms). It includes three phases: (1) presentation of the preliminary (health policy) recommendations, (2) voting on their appropriateness and respective evaluation, and (3) consensus building.

### Blueprint (Work Package 6)

The final project step is to summarize the project findings in a blueprint, including the agreed-upon (health policy) recommendations for actions to reinforce the PE in the context of outpatient surgery (research question 4). The results are classified as general, medical specialty-related (orthopedics), and indication-related. Potential addressees, besides patients, include health care providers, financiers, committees of self-administration in the German statutory health insurance system, as well as legislators. Emphasis is placed on presenting the findings of the project and recommendations in a way that is accessible and understandable for patients. This will be supported through collaboration with the German Health Literacy Network.

### Ethical Considerations

An ethics approval (25‐12629-BO) was obtained from the Ethics Commission of the medical faculty of the University of Duisburg-Essen. Even though registration according to Article 35 of the Declaration of Helsinki is not mandatory, as this is not an interventional study, we registered the study in the German Clinical Trials Register (DRKS00037469). The claims data analysis remains with the TK. The video recordings of the focus groups and interviews will be deleted after transcription. Written informed consent will be obtained from all participants prior to their participation in the focus groups and interviews. For the quantitative questionnaire survey, informed consent was waived by the Ethics Committee due to the fully anonymous nature of the data collection. During transcription, the conversations will be pseudonymized. The final data (information obtained from transcripts and quoted text passages) used for publications can be considered anonymized, as it will no longer be possible to draw any conclusions about the identity of the respondents. All identifying data concerning the patients will be deleted after the focus groups and interviews have been conducted. This also includes the deletion of the audio-visual recordings of the discussions after transcription. An expense allowance is calculated for focus group participation. To take part in workshops, participants will be asked to sign a declaration of consent. Participation in the quantitative survey will remain anonymous. An expense allowance of €100 ($116.95) per participant is planned as an incentive for the time required for the focus groups or interviews.

## Results

The study started in April 2025 and will run until March 2028. As of April 2026, the claims data analysis and the scoping review have been completed. The results of the review are planned to be submitted to a scientific journal in April 2026. The focus groups and interviews were conducted with 19 health care providers and 26 patients and are currently in the data analysis phase and will be completed in May 2026. Subsequently, a manuscript will be submitted by October 2026.

The questionnaire survey, quantifying the results obtained in the basic work packages, will be conducted between May 2026 and March 2027. The submission of results is planned for August 2027.

The workshops are scheduled for the second and fourth quarters of 2027. Finally, the project results will be summarized in a blueprint with recommendations for action to improve PE in the context of orthopedic outpatient surgery by March 2028.

## Discussion

### Principal Findings

Our study will provide both a comprehensive overview of existing problems and barriers to PE in the context of outpatient orthopedic surgery, as well as corresponding solutions or improvements. By including both patients and health care providers, the most relevant perspectives on the topic will be depicted.

The scoping review creates a robust knowledge base by summarizing and mapping international evidence. This knowledge was systematized into different categories, providing a solid foundation to build upon for the project.

The focus groups and interviews discuss the preliminary findings with patients and health care professionals. Thereby, the findings are critically reviewed and supplemented. Here, it is expected that patients can mainly contribute based on their experiences and potential fears. Health care professionals can point out possible limitations or barriers to supporting PE.

The claims data analysis provides an extensive overview of orthopedic indications for which outpatient surgery is a valid option. In this context, frequencies and additional characteristics, such as the ratio between inpatient and outpatient surgeries and sociodemographics, were investigated for each indication of interest.

The questionnaire survey with patients provides a quantification of the preliminary results and assesses the relative importance of individual problems and the suitability of solutions or optimizations. The planned subgroup analysis will reveal differences between individual patient groups (eg, age or sex).

Iteratively discussing the project results with experts from different backgrounds enables the project team to critically evaluate the quantitative and qualitative project outcomes. Furthermore, the workshops help refine preliminary or derive further possible solutions for existing problems and assess their suitability and practicality.

Finally, the blueprint will provide an understandable framework for improving PE in the context of outpatient (orthopedic) surgery. Therefore, it answers the 4 underlying questions of the research project. A possible structure might be (1) challenges and hurdles for patients in the context of outpatient orthopedic surgery, (2) measures for improvement of PE, (3) special considerations for different patient groups, and (4) (health policy) recommendations for action on PE measures in the context of outpatient surgery. Furthermore, the blueprint might contain suggestions for additional patient-relevant materials (eg, information flyers, checklists).

### Comparison to Prior Work

Irrespective of the distinct setting, surgery can lead to consequences such as pain [19] or nausea and vomiting [20]. Outpatient surgeries are no exception [21]. While the health problems treated are serious in themselves, a major problem lies in the insufficient preparation of the postoperative period [22-24], which is essential for the recovery process.

In outpatient surgery, patients must take more responsibility for the postoperative period, as there is no or only limited care and monitoring by medical staff compared to inpatient surgery. To enable patients to take this responsibility, play an active role in their recovery, and manage any problems that arise, it is necessary to increase their PE. This can be achieved by providing information. Here, the content and timing of the information play a key role. As already mentioned in the introduction, too short a period between surgery and information leads to significantly poorer recall of the information conveyed [7]. Regarding the content, too generalized patient information, for example, might lead to too much room for interpretation [24]. Sociodemographic and socioeconomic factors, such as age [25], (health) literacy [26], or the social situation and home environment [25] of patients, could even exacerbate the problem.

While such problems are increasingly recognized, consequences, such as distinct pathways, are still scarce [27]. Therefore, the aim of our project is not only to identify problems and barriers in the context of outpatient surgery, using orthopedics as an example, but also to identify and develop solutions to these problems. Some of the (health policy) recommendations for action might be associated with additional effort. Nevertheless, in addition to the direct patient benefit, they are also likely to make the system more efficient by avoiding longer-term costs (eg, readmission due to postoperative symptoms or unplanned visits to the doctor). Besides only solving the problems in the context of outpatient surgery, the increased PE might additionally leverage their potential benefits.

### Strengths and Limitations

Since this study specifically focuses on Germany and health care systems differ not only in regulation and legislation but also in patient characteristics, the results should always be scrutinized and adapted when transferring them to other systems. In addition to a possible partial transfer to other systems, the results can also be partially applied to other specialist areas. Therefore, the (health policy) recommendations for action will be systematized into “specific for orthopedics” and “universally usable.”

A fundamental strength of our study is the comprehensive integration of patients and their representatives. Both qualitative and quantitative modules include the perspectives of patients. In addition, the project team is advised throughout the project by a patient representative on its procedures and decisions.

### Conclusions

In summary, a high degree of PE in the context of outpatient surgery is essential for ensuring appropriate quality of care and patient satisfaction. The Power-AOP project contributes to this by developing a blueprint, including (health policy) recommendations for action as well as practically relevant, patient-oriented additional material. This helps increase PE by optimizing structures and processes and by providing clear, comprehensible information directly to patients.

## Supplementary material

10.2196/87249 Multimedia Appendix 1 Search strategy MEDLINE via PubMed.

10.2196/87249 Multimedia Appendix 2 Search strategy Embase.

10.2196/87249 Multimedia Appendix 3 Power of the Mann-Whitney *U* test in various scenarios.

## Abbreviations

*ICD-10*: *International Classification of Diseases, Tenth Revision*
PE: patient empowerment
PRISMA-ScR: Preferred Reporting for Systematic Reviews and Meta-Analysis Extension for Scoping Reviews
TK: Techniker Krankenkasse
