# Supplementary material for: Patient Empowerment in the Context of Outpatient Surgery Using the Example of Orthopedics (Power-AOP): Protocol for a Mixed Methods Study
Source: JMIR Res Protoc. 2026 Apr 27;15:e87249. doi: 10.2196/87249 (PMC13117221; doi:10.2196/87249)
Supplement: Multimedia Appendix 1 [file resprot-v15-e87249-s001.docx]

## Appendix 1. Search strategy MEDLINE via PubMed

| (“Ambulatory surgical procedures” [MeSH] OR “Ambulatory surgical procedure*” [tiab] OR“office surger*” [tiab] OR “one-day surger*” [tiab] OR "day surger*" [tiab] OR ((“ambulatory” [tiab] OR “outpatient” [tiab] OR “same-day” [tiab] OR “day-case” [tiab]) AND (“surger*” [tiab] OR “procedure*” [tiab] OR “operation*” [tiab] OR “discharge” [tiab]))) |
| --- |
| AND |
| (“Patient empowerment” [tiab] OR “Patient participation” [MeSH] OR “Patient Participation” [tiab] OR “Patient education” [tiab] OR “Self Care” [MeSH] OR “self-care” [tiab] OR “Information need*” [tiab] OR “Cognitive empowerment” [tiab] OR “Patient know*” [tiab] OR “Patient activation” [tiab] OR “Self-Management” [MeSH] OR “Self-Management” [tiab] OR “Patient cent*” [tiab] OR “Health education” [MeSH] OR “Health education” [tiab] OR “Patient engagement” [tiab] OR “Health literacy” [MeSH] OR “Health literacy” [tiab] OR “self-efficacy” [tiab] OR “person-cent*” [tiab] OR “shared decision making” [tiab] OR “Decision Making, Shared” [MeSH] OR “decision aid*” [tiab] OR “patient preference*” [tiab] OR “patient perception*” [tiab]) |
| AND |
| ((“Orthopedics” [MeSH] OR “Orthopedic*” [tiab] OR “Orthopaedic*” [tiab] OR “Orthopedic procedures” [tiab] OR “fracture*” [tiab] OR “hardware removal” [tiab] OR decompression[tiab] OR discectomy[tiab] OR laminectom*[tiab] OR laminotom*[tiab]) OR ((joint[tiab] OR shoulder[tiab] OR elbow[tiab] OR wrist[tiab] OR hand[tiab] OR hip[tiab] OR knee[tiab] OR ankle[tiab] OR foot[tiab] OR spine[tiab] OR spinal[tiab] OR menisc*[tiab] OR ligament*[tiab]) AND (surgery[tiab] OR surgeries[tiab] OR procedure*[tiab] OR operation*[tiab] OR replacement[tiab] OR arthroplast*[tiab] OR arthroscop*[tiab] OR fusion[tiab] OR fixation[tiab] OR repair[tiab] OR reconstruction[tiab] OR release[tiab] OR osteotom*[tiab]))) |
