# Supplementary material for: Patient Empowerment in the Context of Outpatient Surgery Using the Example of Orthopedics (Power-AOP): Protocol for a Mixed Methods Study
Source: JMIR Res Protoc. 2026 Apr 27;15:e87249. doi: 10.2196/87249 (PMC13117221; doi:10.2196/87249)
Supplement: Multimedia Appendix 2 [file resprot-v15-e87249-s002.docx]

## Appendix 2. Search strategy EMBASE

| ('ambulatory surgery'/exp OR 'day surgery'/exp OR 'ambulatory surgical procedure*':ti,ab OR 'office surger*':ti,ab OR 'one-day surger*':ti,ab OR 'day surger*':ti,ab OR (('ambulatory':ti,ab OR 'outpatient':ti,ab OR 'same-day':ti,ab OR 'day-case':ti,ab ) AND ('surger*':ti,ab OR 'procedure*':ti,ab OR 'operation*':ti,ab OR 'discharge':ti,ab))) |
| --- |
| AND |
| ('patient empowerment'/exp OR 'patient participation'/exp OR 'patient education'/exp OR 'self care'/exp OR 'health education'/exp OR 'self-management'/exp OR 'health literacy'/exp OR 'shared decision making'/exp OR 'self-care':ti,ab OR 'patient participation':ti,ab OR 'patient empowerment':ti,ab OR 'patient education':ti,ab OR 'information need*':ti,ab OR 'cognitive empowerment':ti,ab OR 'patient know*':ti,ab OR 'patient activation':ti,ab OR 'self-management':ti,ab OR 'patient cent*':ti,ab OR 'person-cent*':ti,ab OR 'health education':ti,ab OR 'patient engagement':ti,ab OR 'health literacy':ti,ab OR 'self-efficacy':ti,ab OR 'shared decision making':ti,ab OR 'decision aid*':ti,ab OR 'patient preference*':ti,ab OR 'patient perception*':ti,ab) |
| AND |
| ('orthopedic surgery'/exp OR 'orthopedic procedure'/exp OR 'orthopedic*':ti,ab OR 'orthopaedic*':ti,ab OR 'orthopedic procedures':ti,ab OR fracture*:ti,ab OR 'hardware removal':ti,ab OR decompression:ti,ab OR  discectomy:ti,ab OR laminectom*:ti,ab OR laminotom*:ti,ab OR ((joint:ti,ab OR shoulder:ti,ab OR elbow:ti,ab OR wrist:ti,ab OR hand:ti,ab OR hip:ti,ab OR knee:ti,ab OR ankle:ti,ab OR foot:ti,ab OR spine:ti,ab OR spinal:ti,ab OR menisc*:ti,ab OR ligament*:ti,ab) AND (surgery:ti,ab OR surgeries:ti,ab OR procedure*:ti,ab OR operation*:ti,ab OR replacement:ti,ab OR arthroplast*:ti,ab OR arthroscop*:ti,ab OR fusion:ti,ab OR fixation:ti,ab OR repair:ti,ab OR reconstruction:ti,ab OR release:ti,ab OR osteotom*:ti,ab))) |
