# Supplementary material for: Patient Empowerment in the Context of Outpatient Surgery Using the Example of Orthopedics (Power-AOP): Protocol for a Mixed Methods Study
Source: JMIR Res Protoc. 2026 Apr 27;15:e87249. doi: 10.2196/87249 (PMC13117221; doi:10.2196/87249)
Supplement: Multimedia Appendix 3 [file resprot-v15-e87249-s003.docx]

## Appendix 3. Power of the Mann-Whitney-U-Test in various scenarios

| **n_1_** | **n_2_** | **Effektstärke** | **Power** | **n_1_** | **n_2_** | **Effektstärke** | **Power** |
| --- | --- | --- | --- | --- | --- | --- | --- |
| 1000 | 1000 | 0,2 | 0,997 | 1700 | 300 | 0,5 | 1,000 |
| 1500 | 500 | 0,2 | 0,984 | 1000 | 1000 | 0,8 | 1,000 |
| 1700 | 300 | 0,2 | 0,930 | 1500 | 500 | 0,8 | 1,000 |
| 1000 | 1000 | 0,5 | 1,000 | 1700 | 300 | 0,8 | 1,000 |
| 1500 | 500 | 0,5 | 1,000 |  |  |  |  |
